# Effective Boundary Slip Induced by Surface Roughness and Their Coupled Effect on Convective Heat Transfer of Liquid Flow

**DOI:** 10.3390/e20050334

**Published:** 2018-05-02

**Authors:** Yunlu Pan, Dalei Jing, He Zhang, Xuezeng Zhao

**Affiliations:** 1 Key laboratory of Micro-Systems and Micro-Structures Manufacturing, Ministry of Education and School of Mechatronics Engineering, Harbin Institute of Technology, Harbin 150001, China; 2 School of Mechanical Engineering, University of Shanghai for Science and Technology, Shanghai 200093, China

**Keywords:** surface roughness, effective boundary slip, convective heat transfer

## Abstract

As a significant interfacial property for micro/nano fluidic system, the effective boundary slip can be induced by the surface roughness. However, the effect of surface roughness on the effective slip is still not clear, both increased and decreased effective boundary slip were found with increased roughness. The present work develops a simplified model to study the effect of surface roughness on the effective boundary slip. In the created rough models, the reference position of the rough surfaces to determinate effective boundary slip was set based on ISO/ASME standard and the surface roughness parameters including *Ra* (arithmetical mean deviation of the assessed profile), *Rsm* (mean width of the assessed profile elements) and shape of the texture varied to form different surface roughness. Then, the effective boundary slip of fluid flow through the rough surface was analyzed by using COMSOL 5.3. The results show that the effective boundary slip induced by surface roughness of fully wetted rough surface keeps negative and further decreases with increasing *Ra* or decreasing *Rsm*. Different shape of roughness texture also results in different effective slip. A simplified corrected method for the measured effective boundary slip was developed and proved to be efficient when the *Rsm* is no larger than 200 nm. Another important finding in the present work is that the convective heat transfer firstly increases followed by an unobvious change with increasing *Ra*, while the effective boundary slip keeps decreasing. It is believed that the increasing *Ra* enlarges the area of solid-liquid interface for convective heat transfer, however, when *Ra* is large enough, the decreasing roughness-induced effective boundary slip counteracts the enhancement effect of roughness itself on the convective heat transfer.

## 1. Introduction

The velocity boundary condition of fluid flow in a micro/nano fluidic system with gradually increasing surface to volume ratio can be a significant interfacial property. For the conventional hydrodynamics, it was believed that the velocity of the fluid close to the solid surface was zero, which means there is no relative motion between the solid and fluid at the boundary [1]. However, in the past decades, lots of studies [2,3,4,5,6,7,8] have found that there is a non-zero velocity condition between the solid surface and the adjacent fluid, presenting the so-called boundary slip condition. Although in macro scale, the exist of the boundary slip can be mostly neglected [9,10], there can be lots of potential applications in micro/nano fluidic systems [11,12,13]. The mechanisms of boundary slip are of interest, but there is still no clear understanding of the slip. Previous studies found that surface wettability is a possible reason to affect slip, however, the effect of wettability on slip can be complex and both hydrophobic and hydrophilic surface can induce either slip or no-slip boundary condition, which is dependent on the strength of solid-liquid interface [14,15,16,17,18]. Additionally, surface roughness including texture spacing and texture height can also significantly affect the degree of slip [18,19,20].

Before analyzing the mechanism of boundary slip, two kinds of slip should be distinguished first. The first one is intrinsic boundary slip which represents the directly relative motion between solid and fluid molecular, and the other one is the effective boundary slip which represents the slippage at a “complex heterogeneous” surface evaluated by averaging of a flow over the length scale of the configuration. The understanding of effective slip can be the “calculated equivalent” slip. For example, the solid surfaces cannot be ideally smooth, then due to the roughness, the reference surface position to determinate the effective slip should be at somewhere between the peak and valley. As a result, the boundary condition will be complicated, then, the effective boundary slip should be calculated to describe the liquid flow on the rough surface. In macro scale, if the roughness is small enough, the effective boundary slip can be neglected, while in micro/nano scale, the effective boundary slip induced by the surface roughness may significantly affect the fluid transportation [21].

There were lots of studies on the effect of surface roughness on the effective boundary slip [22,23,24,25,26,27,28,29,30]. However, some of the results showed a larger slip with a larger roughness while some showed opposite. In most of the measurement techniques of boundary slip, such as the atomic force microscopy (AFM) measurement [31,32,33,34] which is believed to be the most accurate one, the surface roughness can affect both of the effective boundary slip and measurement process [30], thus the effective boundary slip induced by the roughness should be clarified.

Besides effective slip, the surface roughness can also affect the contact area of liquid and solid, that is the heat transfer area for convective heat transfer, thus it will finally affect the convective heat transfer of liquid flow, which is quite important in many applications in micro/nano fluidic system and heat exchangers [35,36,37]. Additionally, the roughness-induced boundary slip can also affect the convective heat transfer by changing the flow behavior. Though the effect of boundary slip on the convective heat transfer has been widely studied [38,39,40,41,42,43], the coupled effect of surface roughness and roughness-induced boundary slip on the convective heat transfer is still lacked. The change of the convective heat transfer with different surface roughness should be considered comprehensively.

To solve this problem, in this study, simulation method based on COMSOL 5.3 is first carried out to obtain the relationship between the roughness parameters and the effective boundary slip. Based on the result of surface roughness-induced effective slip, the convective heat transfer property in a rough microchannel with different surface roughness is studied. The underlying mechanisms are discussed.

## 2. Simulation Model

**Figure 1:**
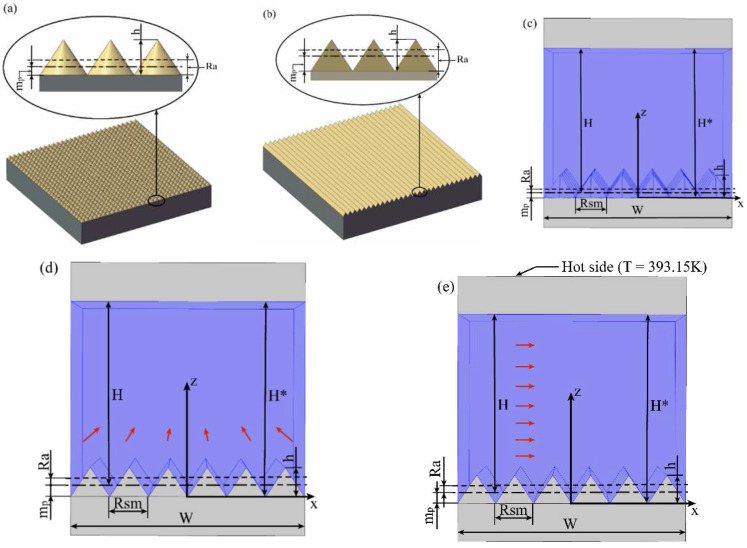
Schematic of the roughness texture in (**a**) the cone model and (**b**) the groove model, and the schematic of the flow on the (**c**) Cone model, (**d**) Groove-P model and (**e**) Groove-V model.

Most of the surface roughness is random texture, which is complicated with lots of parameters. These parameters can be mainly divided into three groups: amplitude parameters, spacing parameters and other parameters, among which, the amplitude and spacing parameters are mostly used. In this study, an amplitude parameter *Ra*, which is the arithmetical mean deviation of the assessed profile of a rough surface, and a spacing parameter *Rsm*, which is the mean width of the profile elements of a rough surface, are chosen to be the key parameters affecting the effective boundary slip. Thus, the simplified models of the surface roughness on flat surface shown in Figure 1, which only contain simple amplitude and pitch characteristic, are used. Figure 1a gives a cone model with two-dimensional roughness, and Figure 1b gives a groove model with one-dimensional roughness. To study the effect of surface roughness on the boundary slip, values of *Ra* and *Rsm* are pre-fixed. Then, the dimensions of roughness textures in the format of cone and groove can be reverse-calculated based on the values of *Ra* and *Rsm* to realize the fixed *Ra* and *Rsm*. It should be noticed that the reference surface position of the rough surface to define *Ra* and *Rsm* is not at the bottom of the profile. Based on ISO/ANSI standard, it should be at some distance above the bottom, the distance *m_p_* of the reference surface position above the bottom can be calculated as [44,45]:

For the cone model:

(1) $$m_{p}=\frac{1}{S}\int_{S}zdxdy=\frac{\pi }{12}h$$

For the groove model:

(2) $$m_{p}=\frac{1}{S}\int_{S}zdxdz=\frac{h}{2}$$

where *S* is the projection area of the texture, *z* is the coordinate along the direction of texture height, and *h* is the height. Then, the dimensions of cone model and the groove model can be respectively reverse-calculated based *Ra* and *Rsm* as follows.

For cone model,

$$r=\frac{1}{2}Rsm,\;h=\frac{10368}{\pi {\left(12-\pi \right)}^{3}}Ra\approx 4.75Ra$$

where *r* is the is the radius of the bottom circle of the cone.

For groove model,

l=Rsm, h=4Ra

where *l* is the width of the groove.

Based on these rough surfaces, pressure driven flow through a straight channel confined by a lower side of the created rough surface and a upper side of a fully smooth surface was simulated. Furthermore, it is assumed that there is no boundary slip on both the lower and upper surfaces. The flow on the cone model is shown as Figure 1c, while for the groove model, two situations are set with different direction of flow on it, one’s flow is parallel with the groove which is labeled groove-p model shown as Figure 1d while the other one’s flow is vertical with the groove which is labeled groove-v model shown as Figure 1e.

Models with different *Ra* and *Rsm* are built to study the relationship between the roughness and boundary slip and their effect on heat transfer. The distance between the upper wall and the bottom profile of the rough lower wall H* is set as 20,000 nm, and the thickness of the two walls is 2000 nm. The external reference pressure is set as 1 bar (1.01325 × 10^5^ Pa), and room temperature is 293.15 K. Then the density of water *ρ* = 0.9982071 g/cm^3^, and dynamic viscosity *μ* = 1.0050 × 10^−3^ Pa⋅s [46]. Based on the setup, the flow field can be assumed to remains laminar. *p_in_* is set as a constant at the inlet of the channel while *p_out_* is set as 0 Pa at the outlet of the channel. Moreover, the lateral sides of the fluid flow field are defined by symmetric plane. Based on the simulation by COMSOL 5.3, the flow rate *Q* can be obtained for each model.

The surface roughness will avoidably affect the flow rate of the fluid flow, and this surface roughness-dependent flow rate can be characterized by the effective boundary slip induced by the surface roughness. Assuming the surface roughness-induced effective slip on the lower rough surface is *b_eff_*, then the relationship between effective slip *b_eff_* and flow rate Q can be described as [47]:

(3) $$b_{eff}=-\frac{12\mu HQ+\left(dp/dx\right)H^{4}W}{4\left(dp/dx\right)H^{3}W+12\mu Q}$$

where d*p*/d*x* is the pressure gradient. Therefore, the effective slip *b_eff_* can be obtained with Equation (3). The height *H* of the channel need to be calculated for each model by *H* = H^*^ − *m_p_* considering the surface roughness.

Then, for the cone model:

(4) $$H=H^{*}-\frac{\pi }{12}h$$

for the groove model:

(5) $$H=H^{*}-\frac{h}{2}$$

The heat transfer model is built by adding the heat transfer properties including convection and conduction to the liquid and solid of the groove-v model, shown as Figure 1e. It is a simulation of a typical water cooling system. The heat source with a constant temperature of 393.15 K is applied on the outside surface of the upper wall of the channel. The flow between the two plates is assumed to be non-isothermal flow, and it will cool the upper plate by convective heat transfer. The other parameter is as same as the previous simulation. The *Rsm* of the rough surface is fixed at 100 nm while *Ra* is variable. Based on the simulation by COMSOL 5.3, the bulk convective heat transfer power *P* can be obtained, which is related to the Nusselt number as shown in Equation (6).

(6) $$Nu=\frac{PH}{kA\Delta T}$$

where *k* is the thermal conductivity of the fluid, *A* is the surface area, ΔT is the temperature difference.

## 3. Results and Discussion

Based on the created models, the effective boundary slip on surfaces with different *Ra* and *Rsm* are obtained. Then the convective heat transfer performance of the pressure-drive flow is obtained on groove model with different *Ra*. To keep the accuracy of the present analysis using COMSOL 5.3, the grid sensitivity is carefully checked. Through the grid sensitivity analysis, the grid in free tetrahedral shape is used and the number of gird is about 2 × 10^5^. The results are shown as following.

### 3.1. Effective Boundary Slip

**Figure 2:**
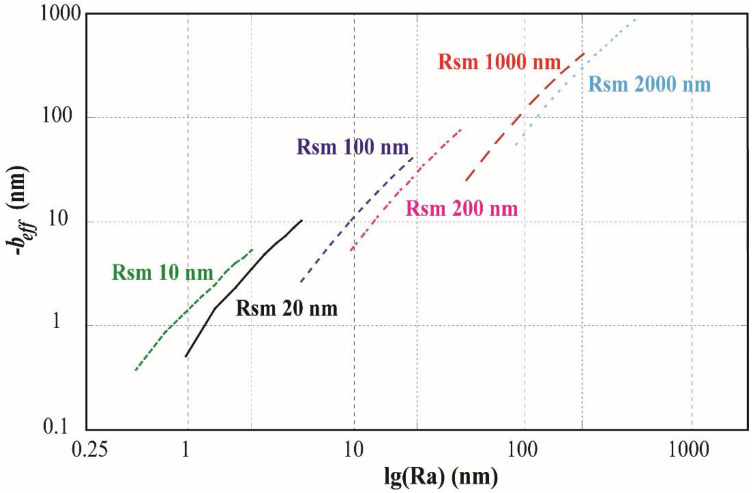
Effective boundary slip (reversed) vs. *Ra* on the cone model with *Rsm* = 10 nm (Green); 20 nm (Black); 100 nm (Dark blue); 200 nm (Pink); 1000 nm (Red); 2000 nm (Light blue). The −*b_eff_* and *Ra* are in log scale.

The effective boundary slip on the cone model with different *Ra* and *Rsm* are shown in Figure 2. Form the results in Figure 2, it can be found that the effective boundary slip induced by the surface roughness is always negative, which means the surface roughness of the microchannel wall in the present work always reduces the flow rate and increases the fluid drag compared to a microchannel confined by two fully smooth walls without slip. Additionally, it is obvious that with the same *Rsm*, increasing *Ra* will lead to a decrease of the effective boundary slip and with same *Ra*, increasing *Rsm* will lead to an increase of the effective boundary slip.

**Figure 3:**
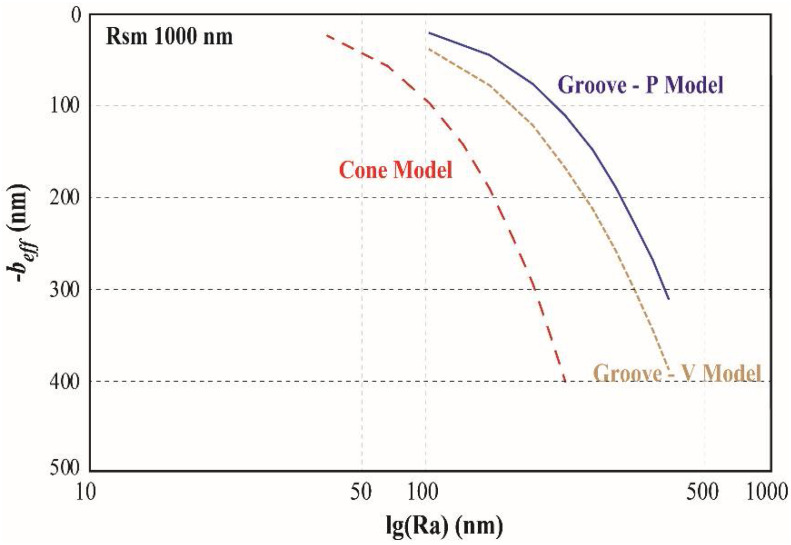
Effective boundary slip (reversed) vs. *Ra* on three different surface models: Cone model; Groove-V model and Groove-P model. The *Ra* is in log scale.

In the two groove models, the trends of effective boundary slip with *Ra* and *Rsm* are similar with that in cone model. However three different models have different slips. The effective boundary slips with the same *Rsm* of 1000 nm on three different models are shown in Figure 3. It is shown that firstly the slip has the same trend with *Ra* on three models, and secondly the groove models have the larger slip than cone model.

As a summary, the effective boundary slip induced by the roughness of the rough surface with zero intrinsic boundary slip is always negative, and it will decrease on rougher surface and it also depends on the shape of the surface roughness. This means that the surface roughness in the present work always increases the fluid drag and the fluid drag increase with the increasing roughness. These results are in agreement with the previous studies [28,29,31,48,49,50], and all these theoretical and experimental studies found that friction factor increased with the growing surface relative roughness.

Based on the present work regarding effect of surface roughness on effective boundary slip, the effective boundary slip is related to the surface roughness and the reference surface of the rough surface, thus, most of experimental measurement of slip on rough surface is not the real effective boundary slip and should be corrected. Correction methods were developed by groups [29,30]. Based on definition from ISO/ANSI, the mean plane should be used as the reference position with no doubt. However, in AFM measurement of boundary slip, the reference position was always set at the contact position of the probe and surface. The contact position should be at the top point of the surface morphology at the contact area, since there is a roughness parameter called *Rpm* which is defined as the distance between the average peak and the mean plane, then the correction can be simply subtract the *Rpm* from the measured boundary slip.

**Figure 4:**
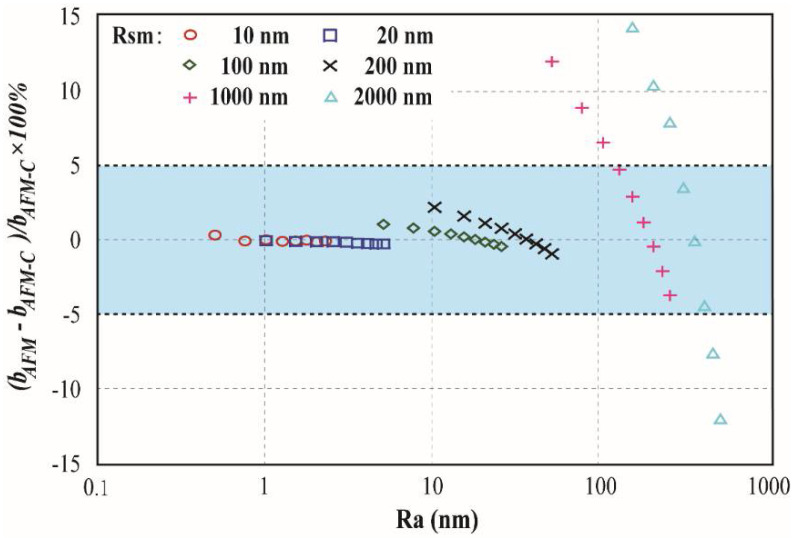
Error of corrected effective boundary slip (obtained experimentally from AFM) on cone model with different *R*_a_ and *R*_sm_.

To check the validity of the correction method, in this study, the reference surface position was set at the top point of surface morphology to simulate the slip obtained experimentally with AFM, labeled as *b_AFM_*. Then the results were corrected by simply subtract the distance between the two different reference surface position which is referred to the *R*_pm_ of the surface. The corrected effective boundary slip is marked as *b_AFM-C_*. Then the error of the corrected slip are obtained and shown in Figure 4. In Figure 3, it is shown that with smaller *Rsm*, the errors are always smaller than ±5% which is acceptable for slip measurement. However, when the *Rsm* reaches 1000 nm or even 2000 nm, the error can be as large as 15%. Error as 15% for boundary slip measurement seems to be too large, however, it should be noticed that the error of the uncorrected experimentally measured slip is in range of 60–600%, as a result, the correction is still necessary and the error is acceptable, especially when *Rsm* is smaller than 200 nm.

### 3.2. Convective Heat Transfer

**Figure 5:**
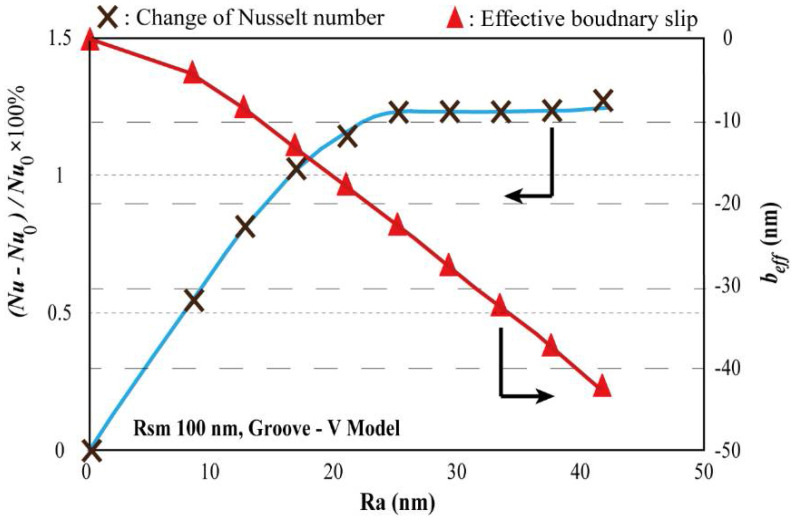
Increasing rate of Nusselt number for a pressure-driven flow in a micro channel with one-side rough surface in Groove-V model. The *Rsm* is fixed at 100 nm. The effective boundary slip are also shown.

The bulk convective heat transfer is obtained on the groove-v model. For easier understanding, the bulk convective heat transfer is characterized by the Nusselt number *Nu*. Nusselt number for the ideally smooth surface is written as *Nu*_0_. The variation of Nusselt number with different *Ra* is shown in Figure 5. From Figure 5, it can be found that when the *Ra* increases from 0, the Nusselt number increases faster when *Ra* is lower than 25 nm, followed by a slow and unobvious increase. The increasing Nusselt number with the increasing *Ra* when *Ra* is small is in well agreement with the previous theoretical and experimental studies [48,49,50], this is because that the increase of *Ra* extends the real contact area of solid and liquid, which can enhance the convective heat transfer. In the meanwhile, the effective boundary slip decreases continually. However, when *Ra* is larger enough, the decreased effective boundary slip induced by surface roughness will lead to a significant reduction of the liquid flow velocity, which will weaken the convective heat transfer. The enhancement of *Ra* on the heat transfer by increasing the real contact area of solid and liquid and the reduction of *Ra* on the heat transfer by decreasing the effective slip counteract each other.

## 4. Conclusions

In this paper, the effective boundary slip induced by the surface roughness is studied with different *Ra* and *Rsm* based on a simplified model by a quantitative simulation. The convective heat transfer power of the pressure driven flow on rough surfaces are studied in the same model as well. The results show that for totally wetting rough surfaces with zero slip, the effective boundary slip induced by the roughness is always negative which means the surface roughness will always increase the drag of liquid flow at the interface, in the meanwhile, the negative effective boundary slip will further decrease with increasing *Ra* or decreasing *Rsm.* It is also found that the shape of the roughness texture will affect the effective boundary slip. For the surfaces with smaller *Rsm*, the simple corrected method for the measured slip by AFM, which subtracts the distance between two reference surface positions from the final results, can effectively decrease the error from up to 600% to 5%. Furthermore, the convective heat transfer can be enhanced with larger *Ra* of the surface, however, the increase of the convective heat transfer or Nusselt number will be unobvious when the *Ra* keep increasing due to the decreased effective boundary slip.
